# NMR-Based Metabolomic Investigations on the Differential Responses in Adductor Muscles from Two Pedigrees of Manila Clam *Ruditapes philippinarum* to Cadmium and Zinc

**DOI:** 10.3390/md9091566

**Published:** 2011-09-19

**Authors:** Huifeng Wu, Xiaoli Liu, Jianmin Zhao, Junbao Yu

**Affiliations:** 1 Key Laboratory of Coastal Zone Environment Processes, CAS; Shandong Provincial Key Laboratory of Coastal Zone Environment Processes, Yantai Institute of Coastal Zone Research, Chinese Academy of Sciences, Yantai 264003, China; E-Mails: lxlshz2006@163.com (X.L.); jmzhao@yic.ac.cn (J.Z.); jbyu@yic.ac.cn (J.Y.); 2 The Graduate School of Chinese Academy of Sciences, Beijing 100049, China

**Keywords:** *Ruditapes philippinarum*, heavy metal, biomonitor, NMR, metabolomics

## Abstract

Manila clam *Ruditapes philippinarum* is one of the most important economic species in shellfishery in China due to its wide geographic distribution and high tolerance to environmental changes (e.g., salinity, temperature). In addition, Manila clam is a good biomonitor/bioindicator in “Mussel Watch Programs” and marine environmental toxicology. However, there are several pedigrees of *R. philippinarum* distributed in the marine environment in China. No attention has been paid to the biological differences between various pedigrees of Manila clams, which may introduce undesirable biological variation in toxicology studies. In this study, we applied NMR-based metabolomics to detect the biological differences in two main pedigrees (White and Zebra) of *R. philippinarum* and their differential responses to heavy metal exposures (Cadmium and Zinc) using adductor muscle as a target tissue to define one sensitive pedigree of *R. philippinarum* as biomonitor for heavy metals. Our results indicated that there were significant metabolic differences in adductor muscle tissues between White and Zebra clams, including higher levels of alanine, glutamine, hypotaurine, phosphocholine and homarine in White clam muscles and higher levels of branched chain amino acids (valine, leucine and isoleucine), succinate and 4-aminobutyrate in Zebra clam muscles, respectively. Differential metabolic responses to heavy metals between White and Zebra clams were also found. Overall, we concluded that White pedigree of clam could be a preferable bioindicator/biomonitor in marine toxicology studies and for marine heavy metals based on the relatively high sensitivity to heavy metals.

## 1. Introduction

Metabolomics is the “systematic study of the unique chemical fingerprints that specific cellular processes leave behind” [1]. Basically, such a study focuses on the global analysis of all the low molecular weight (<1000 Da) metabolites which are the end products of metabolism, representing the functional responses in biological systems (e.g., cell, tissue, urine, plasma) [2,3]. Due to its high throughput and low expenditure, metabolomics has been successfully used in drug toxicity, disease diagnosis, functional genomics, and environmental sciences [4–8]. There are several modern analytical techniques, especially mass spectrometry (MS) and nuclear magnetic resonance (NMR) spectroscopy, are widely applied in metabolomics [9–11]. High resolution proton nuclear magnetic resonance (HR-^1^H NMR) spectroscopy is uniquely suited to detect a large range of endogenous low molecular weight metabolites in an organism, since this technique is rapid and rich in structural and quantitative information and allows the metabolites to be analyzed simultaneously [2,4]. It is well established that NMR techniques combined with computer-based pattern recognition methods can provide insightful biological and biochemical information on the biochemical perturbations that are induced by both endogenous and exogenous factors, through the analysis of biofluids and tissues [2,12]. To date, NMR-based metabolomics has been efficiently used in marine environmental sciences [3,7,8,13–18].

Manila clam *Ruditapes philippinarum* is an economic species in shellfishery in China because of its wide geographic distribution, high tolerance to environmental changes (e.g., salinity, temperature) and great consumption as seafood. In addition, Manila clam is a good biomonitor/bioindicator in “Mussel Watch Programs” and marine environmental toxicology [19–23]. However, there are several pedigrees (White, Zebra, Liangdao Red and Marine Red) of *R. philippinarum* distributed in the marine environment in China. No attention has been paid to the biological differences between various pedigrees of Manila clams, which may introduce undesirable variation in marine environmental toxicology. Some evidences indicated that different pedigrees of clams had differential tolerances to environmental stressors [24,25]. Yan *et al.* reported that Zebra pedigree of clam had the highest survival rate and tolerance to environmental stressors (e.g., high temperature) than other pedigrees [24]. Clearly, it is necessary to assess the biological difference between various pedigrees of Manila clam and differential responses to marine environmental heavy metal contaminants.

We previously investigated the differential metabolic responses in gill tissues from various pedigrees of clams exposed to mercury and concluded that White clam was the preferable bioindicator for marine mercury monitoring [26]. In this study, two typical heavy metal contaminants, cadmium (Cd) and zinc (Zn), were used as non-essential and essential metals for exposures to two main pedigrees (White and Zebra) of Manila clam. Cd and Zn are commonly found as contaminants in the Bohai marine environment [27,28]. Although adductor muscle can accumulate less amounts of heavy metals than other organs, such as digestive gland and gills because of its solidness, it contains much less lipids as well [29]. Lipids are usually undesirable macromolecules that can overlap other low molecular weight metabolites due to the broad peaks in proton NMR spectrum [30,31]. Therefore the adductor muscle of Manila clam was chosen as a target tissue in this work. Here we need to answer three questions. Firstly, are there significant biological differences in adductor muscles between White and Zebra clams based on the metabolic profiles? Secondly, are the metabolic responses in adductor muscles different between White and Zebra clams to heavy metal exposures (Cd, Zn and mixed Cd and Zn)? Thirdly, which pedigree is more sensitive to heavy metal contaminants, Cd and Zn, when adductor muscle is used as a target tissue? In order to answer these three questions, the metabolic differences in adductor muscles between White and Zebra clams and the differential responses between the control and heavy metal-exposed groups were determined by NMR-based metabolomics.

## 2. Results and Discussion

### 2.1. Metabolic Differences in Adductor Muscles between White and Zebra Clams

**Figure 1:**
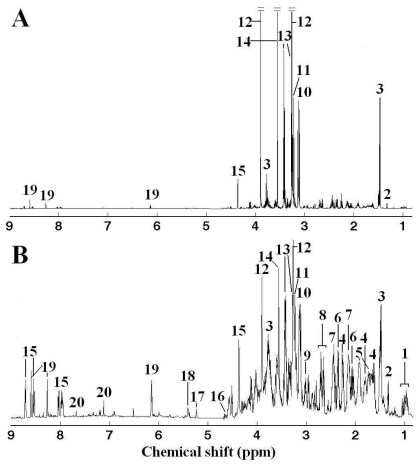
A representative 1-dimensional 500 MHz ^1^H NMR spectrum of adductor muscle extracts from a White clam of control group in original (**A**) and generalized log transformed (λ = 5.0 × 10^−10^) (**B**) forms. Keys: (1) branched chain amino acids: isoleucine, leucine and valine, (2) lactate, (3) alanine, (4) 2-aminoadipate, (5) arginine, (6) glutamate, (7) glutamine, (8) aspartate, (9) 4-aminobutyrate, (10) unkown 1 (3.14 ppm), (11) phosphocholine, (12) betaine, (13) taurine, (14) glycine, (15) homarine, (16) β-glucose, (17) α-glucose, (18) glycogen, (19) ATP and (20) histidine.

A representative ^1^H NMR spectrum of adductor muscle tissue extracts from a White clam is shown in original (Figure 1A) and generalized log (glog) transformed (Figure 1B) forms in Figure 1. Several metabolite classes were identified, including amino acids (branched chain amino acids: valine, leucine and isoleucine, aspartate, glutamate, glycine, *etc*.), energy storage compounds (ATP, glucose and glycogen) and osmolytes (betaine, taurine and homarine). As observed, the original NMR spectrum (Figure 1A) is dominated by several organic osmolytes, betaine (3.27 and 3.91 ppm), taurine (3.27 and 3.45 ppm), glycine (3.57 ppm) and alanine (1.48 ppm) (Figure 1A), which is approx. 10 times intense than other metabolites. Organic osmolytes such as betaine, homarine, and taurine are small organic molecules to regulate the osmotic balance in marine organisms. These osmolytes can be effectively accumulated in clams to high environmental salinity and released when the salinity decreases. Although free amino acids such as glycine and alanine are mainly involved in the metabolism of proteins, recent studies have reported that some marine mollusks used high intracellular concentrations of free amino acids to balance their intracellular osmolarity with the environment [32,33]. Therefore, some amino acids play important role in osmotic regulation in *R. philippinarum* and were detected at high level in adductor muscles, together with other osmolytes including betaine, taurine and homarine [33].

**Figure 2:**
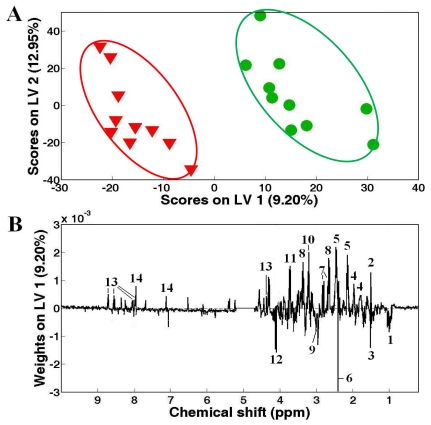
Partial least squares-discriminant analysis (PLS-DA) model showing (**A**) separations between control White ( 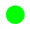) and Zebra ( 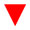) clam samples, and corresponding LV1 (**B**) weights plot showing the metabolic differences between White and Zebra pedigrees of clam adductor muscle tissue extracts. Keys: (1) branched chain amino acids, (2) alanine, (3) unknown 1 (1.51 ppm), (4) arginine, (5) glutamine, (6) succinate, (7) aspartate, (8) hypotaurine, (9) 4-aminobutorate, (10) phosphocholine, (11) unknown 2 (3.72 ppm), (12) unknown 3 (4.12 ppm), (13) homarine and (14) histidine.

The unsupervised pattern recognition method, principal component analysis (PCA), was initially applied to the NMR spectral datasets of adductor muscle extracts from control White (green cycles) and control Zebra (inverted red triangles) clam samples to compare the metabolic profiles. However, no significant separation (*p* > 0.05) was found between the groups using PCA (data not shown), then the supervised pattern recognition technique, partial least square-discriminant analysis (PLS-DA) was used to classify the two pedigrees of clam samples (Figure 2). PLS-DA resulted in a clear separation between White and Zebra clam samples with a high *Q*^2^ value greater than 0.4 (Figure 2A). The potential metabolic differences between the metabolic profiles were shown in the weights plot of first latent variable (LV1) axis (Figure 2B). After one way analysis of variance (ANOVA) combined with a false discovery rate (FDR) at 0.01, the significantly different metabolites were identified and labeled in LV1 weights plot (Figure 2B). Basically, there were several abundant metabolites including alanine, arginine, glutamine, hypotaurine, phosphocholine and homarine in adductor muscle tissue from White clams (Figure 2B). The metabolic profile of Zebra clam samples showed high levels of branched chain amino acids, succinate and 4-aminobutyrate compared with that of White clam samples. Since both White and Zebra clams are from the same species *R. philippinarum* sharing the similar *genotypic milieu*, the differential phenotypic fingerprints (e.g., metabolic differences) might be generated from the differential gene expressions and consequent amounts of enzymes related to various metabolisms. For example, two osmolytes, hypotaurine and homarine were more abundant in White clam adductor muscle, which implied that White clam could use more hypotaurine and homarine to balance the cellular osmolarity to environment. However, Zebra clam might mobilize high levels of branched chain amino acids to maintain osmotic balance, which was observed in other marine mollusks [32].

### 2.2. Differential Responses in Adductor Muscles from White and Zebra Clams to Heavy Metal Exposures

**Figure 3:**
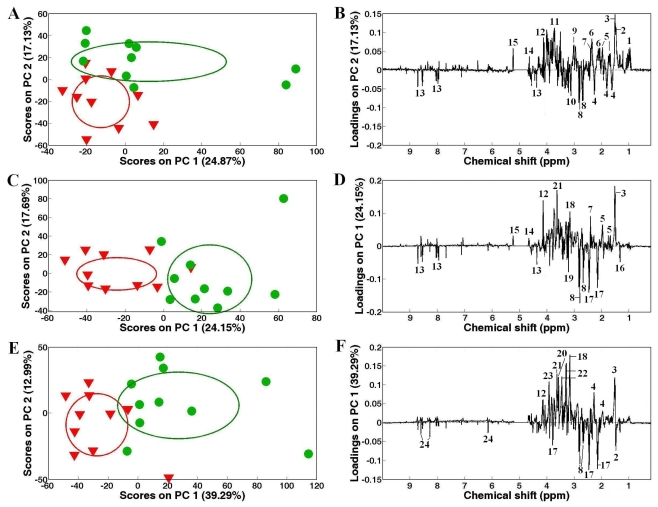
Principal components analysis (PCA) on the ^1^H NMR spectra showing significant separations between control ( 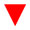) and Cd (**A**), Zn (**C**) and mixed Cd and Zn (**E**) exposed ( 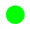) White clams, and corresponding PC loadings plots, (**B**), (**D**) and (**F**) showing the metabolic differences between the control and heavy metal-exposed clam samples after exposure for 48 h. Ellipses represented mean ± standard deviation of PC scores for each group. Keys: (1) branched chain amino acids, (2) alanine, (3) unknown 1 (1.51 ppm), (4) 2-aminoadipate, (5) arginine, (6) glutamate, (7) succinate, (8) aspartate, (9) 4-aminobutyrate, (10) unknown 2 (3.14 ppm), (11) unknown 3 (3.72 ppm), (12) unknown 4 (4.12 ppm), (13) homarine, (14) β-glucose, (15) α-glucose, (16) lactate, (17) glutamine, (18) unknown 4 (3.16 ppm), (19) phosphocholine, (20) glycine, (21) unknown 5 (3.63 ppm), (22) taurine, (23) betaine and (24) ATP.

**Figure 4:**
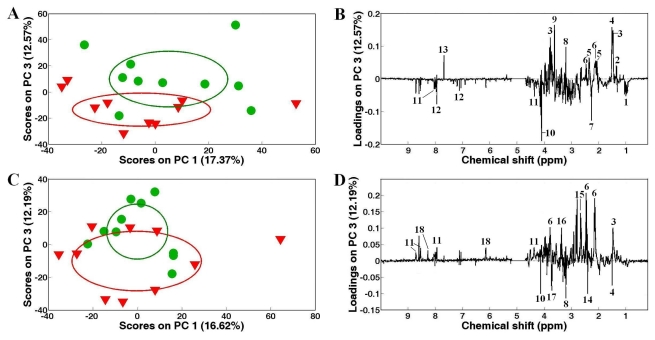
Principal components analysis (PCA) on the ^1^H NMR spectra showing significant separations between control ( 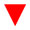) and Cd (**A**), Zn (**C**) exposed ( 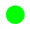) Zebra clams, and corresponding PC loadings plots, (**B**), (**D**) showing the metabolic differences between the control and heavy metal-exposed clam samples after exposure for 48 h. Ellipses represented mean ± standard deviation of PC scores for each group. Keys: (1) branched chain amino acids, (2) lactate, (3) alanine, (4) unknown 1 (1.51 ppm), (5) glutamate, (6) glutamine, (7) acetoacetate, (8) phosphocholine, (7) aspartate, (9) unknown 2 (3.63 ppm), (10) unknown 3 (4.12 ppm), (11) homarine, (12) histidine, (13) unknown 4 (7.68 ppm), (14) succinate, (15) aspartate, (16) hypotaurine, (17) unknown 5 (3.72 ppm) and (18) ATP.

PCA was performed on the ^1^H NMR spectral data sets generated from the control and heavy metal (Cd, Zn and Cd + Zn) exposed groups of clams from White and Zebra pedigrees, respectively (Figures 3 and 4). For the White clam samples, the control (inverted red triangles) and heavy metal exposed groups (green cycles) were highly significantly (*P* < 0.01) separated along various PC axes (Figure 3A,C,E). The significant metabolic biomarkers induced by Cd in White clam adductor muscles included the elevated branched chain amino acids (valine, leucine and isoleucine), alanine, arginine, glutamate, succinate, 4-aminobutyrate and glucose, together with the depleted 2-aminoadipate, aspartate and homarine. In Zn-treated White clam muscles, significantly increased arginine, succinate, glycine and glucose were found as well as decreased lactate, glutamine, aspartate, phosphocholine and homarine. For the mixed exposure of Cd and Zn, the detectable metabolic responses comprised the up-regulated 2-aminoadipate, taurine, glycine and betaine, and down-regulated alanine, glutamine, aspartate, and ATP in White clam muscles.

**Figure 5:**
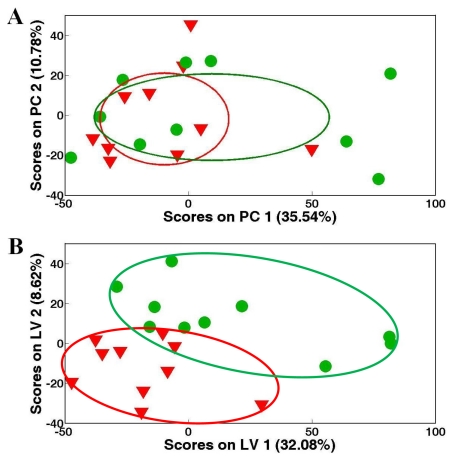
Principal components analysis (**A**) and partial least squares-discriminant analysis (**B**) on the ^1^H NMR spectra from control and mixed Cd and Zn exposed Zebra clam samples. Note: In (**A**), ellipses represented mean ± standard deviation of PC scores for each group.

Based on the PC scores plots (Figure 4A,C), the separations between control (inverted red triangles) and Cd or Zn treated Zebra clam samples (green cycles) were significant (*P* < 0.05). However, PCA was performed on the data sets from Cd + Zn exposed and control Zebra clam samples resulting in no significant (*p* = 0.98) separation (Figure 5A). PLS-DA was then employed to classify control and mixed heavy metal exposed Zebra clam samples (Figure 5B). However, the PLS-DA model was not robust and reliable due to the low *Q*^2^ value (<0.1) and high classification error (0.55) of cross validation. These findings implied that both Cd and Zn could induce significant metabolic responses in Zebra clam muscle, whilst the mixed exposure of Cd and Zn caused no obvious metabolic changes in Zebra clam muscles. It clearly indicated the antagonistic effects of Cd and Zn in Zebra clam adductor muscles. To identify the significant metabolites in Cd and Zn treated Zebra clam samples, one way ANOVA was conducted on the ratio of bin areas to the total spectral area combined with false discovery rate at 0.01. The significant metabolic responses to Cd exposure included the increased lactate, alanine, glutamate, glutamine and phosphocholine in Zebra clam adductor muscles as well as the decreased branched chain amino acids, acetoacetate, homarine and histidine. Zinc exposure induced clear increases in alanine, glutamine, aspartate, hypotaurine, ATP and histidine, and decreases in succinate and phosphocholine (Figure 4B,D).

Heavy metal exposures (Cd and Zn) induced significant metabolic changes in both White and Zebra clam muscles. However, the altered metabolic profiles differed between White and Zebra clam samples exposed to Cd or Zn. Besides the similar metabolic responses including the increased alanine and glutamate and decreased homarine, some contrarily altered metabolites such as branched chain amino acids (valine, leucine and isoleucine) were found in either White or Zebra clam samples exposed to Cd. Uniquely, elevated arginine, succinate, 4-aminobutyrate and glucose and depleted 2-aminoadipate and aspartate were discovered in Cd-treated White clam samples as well as elevated glutamine and phosphocholine and depleted acetoacetate and histidine in Cd-treated Zebra clam samples. These distinctive metabolic biomarkers between White and Zebra clam samples meant differential toxicological effects in White and Zebra clam samples. For example, elevated phosphocholine indicated Cd-induced disturbance in energy metabolism in Zebra clam samples, which was not found in White clam samples [7]. For Zn exposure, the metabolic profiles between White and Zebra clam samples showed dissimilar metabolic changes except depleted phosphocholine. The mechanisms were unclear; however, these differential responsive mechanisms suggested that one pure pedigree of clams should be used in marine toxicology study. After exposure with mixed Cd and Zn, the metabolic responses were somewhat similar to those of Cd- and Zn-treated samples from White clams, however, unique metabolic changes including increased taurine and betaine and decreased ATP were found in mixed Cd and Zn-treated White clam adductor muscles, which indicated the synergistic effects induced by Cd and Zn in White clam adductor muscles. Overall, White clam was more sensitive than Zebra clam to heavy metal exposures (Cd, Zn and mixed Cd and Zn) since more metabolites were sensitively inducible in White clams when adductor muscle was used as a target tissue.

## 3. Experimental

### 3.1. Chemicals

Sodium dihydrogen phosphate (Na_2_HPO_4_), disodium hydrogen phosphate (NaH_2_PO_4_), cadmium chloride (CdCl_2_) and zinc chloride (ZnCl_2_) (all in analytical grade) were purchased from Guoyao Chemical Co. Ltd. (Shanghai, China). Extraction solvents, methanol and chloroform (HPLC grade) were purchased from Guoyao Chemical Co. Ltd. (Shanghai, China). Deuterium oxide (D_2_O, 99.9% in D) and sodium 3-trimethlysilyl [2,2,3,3-D4] propionate (TSP) were purchased from Cambridge Isotope Laboratories (Miami, FL, USA).

### 3.2. Clam Exposure

Eighty adult clams *R. philippinarum* (shell length: 3.4–3.8 cm, *n* = 40 from White and Zebra pedigrees, respectively) were purchased from local culturing farm. The clams were allowed to acclimate in aerated seawater (25 °C, 33 psu, collected from pristine environment) in the laboratory for 1 week and fed with the *Chlorella vulgaris* Beij at a ratio of 2% tissue dry weight daily. After acclimatization, the clams were randomly divided into four tanks (one control and three heavy metal exposures). Each tank contained 10 White and 10 Zebra clams which were exposed to dissolved Cd^2+^ (20 μg L^−1^), Zn^2+^ (50 μg L^−1^), or a mixture of Cd^2+^ (20 μg L^−1^) and Zn^2+^ (50 μg L^−1^) for 48 h. Cadmium and zinc were prepared from CdCl_2_ and ZnCl_2_ (analytical grades). The experimental concentrations of Cd^2+^ and Zn^2+^ can be found in heavily polluted sites along the Bohai Sea [27,28]. After 48 h of exposure, all the clams were immediately dissected for adductor muscles which were flash frozen in liquid nitrogen, and then stored at −80 °C before metabolite extraction (*n* = 10).

### 3.3. Metabolite Extraction

Polar metabolites in adductor muscles of Manila clams were extracted by the modified extraction protocol as described previously [15,26,30,31,34]. Briefly, the adductor muscle (*ca.* 100 mg) was homogenized and extracted in 4 mL g^−1^ of methanol, 5.25 mL g^−1^ of water and 2 mL g^−1^ of chloroform. The methanol/water layer with polar metabolites was transferred to a glass vial and dried in a centrifugal concentrator. The extracts of adductor muscle tissue were subsequently re-suspended in 600 μL of 100 mM of phosphate buffer (Na_2_HPO_4_ and NaH_2_PO_4_, including 0.5 mM TSP, pH 7.0) in D_2_O. The mixture was vortexed and then centrifuged at 3000 g for 5 min at 4 °C. The supernatant substance (550 μL) was then pipetted into a 5 mm NMR tube prior to NMR analysis.

### 3.4. High Resolution One Dimensional ^1^H NMR Spectroscopy

Extracts of adductor muscle from clams were analyzed on a Bruker AV 500 NMR spectrometer performed at 500.18 MHz (at 298 K) as described previously [26,34]. Briefly, one-dimensional ^1^H NMR spectra were obtained using a 11.9 μs pulse, 6009.6 Hz spectral width, mixing time 0.1 s, and 3.0 s relaxation delay with standard 1D NOESY pulse sequence to suppress the residual water peak, with 128 transients collected into 16,384 data points. Datasets were zero-filled to 32,768 points, and exponential line-broadenings of 0.3 Hz were applied before Fourier transformation. All ^1^H NMR spectra were phased, baseline-corrected, and calibrated (TSP at 0.0 ppm) manually using TopSpin (version 2.1, Bruker). NMR spectral peaks were assigned following tabulated chemical shifts [32,35] and using the software, Chenomx (Evaluation Version, Chenomx Inc., Canada).

### 3.5. Spectral Pre-Processing and Multivariate Data Analysis

One dimensional proton NMR spectra were converted to a format for multivariate analysis using custom-written ProMetab software in Matlab (version 7.0; The MathsWorks, Natick, MA) [36]. Each spectrum was segmented into 0.005 ppm bins between 0.2 and 10.0 ppm with bins from 4.70 to 5.20 ppm (water) excluded from all the NMR spectra. The total spectral area of the remaining bins was normalized to unity to facilitate the comparison between the spectra. All the NMR spectra were generalized log transformed (glog) with transformation parameter λ = 5.0 × 10^−10^ [36,37] to stabilize the variance across the spectral bins and to increase the weightings of the less intense peaks. Data were mean-centered before principal components analysis (PCA) and partial least squares-discriminant analysis (PLS-DA) using PLS Toolbox (version 4.0, Eigenvector Research, Manson, WA).

Two well-developed pattern recognition techniques, PCA and PLS-DA, were used in this work for the separation of sample groups. PCA is an exploratory unsupervised pattern recognition method of analysis which is blind to the status of each sample, and serves to reduce the dimensionality of the data and summarize the similarities and differences between multiple NMR spectra [38]. The algorithm of this pattern recognition method calculates the highest amount of correlated variation along PC1, with subsequent PCs containing correspondingly smaller amounts of variance. For each model built, the loading vector for the PC was examined to identify the metabolites which contributed to the clusters. One way analysis of variance (ANOVA) was conducted on the PC scores from each group to test the statistical significance (*p* < 0.05) of separations. PLS-DA is a supervised pattern recognition method and is used to classify 2 or more classes, by searching for variables (X matrix) which are correlated to class membership (Y matrix). In PLS-DA, the X matrix is the measured matrix, *i.e.*, the NMR data, and the Y matrix is made of dummy variables consisting of ones and zeros that indicate the class for each treatment [39]. The quality of the PLS-DA model was assessed using cross-validation with five-way split Venetian blinds [40]. A *Q*^2^ score greater than 0.4 indicates that the model is practically robust [41]. SAM software was then used to find significant metabolic differences various groups with appropriate false discovery rate (FDR < 0.01) cutoffs [42]. These significant metabolites were contributive for the separation between control and heavy metal treated samples and hence were considered metabolic biomarkers induced by heavy metal exposures. A *p* value of 0.05 was considered significant for the ANOVA on the metabolites between control and exposed groups.

## 4. Conclusions

This study focused on the biological difference between two pedigrees (White and Zebra) of Manila clam *Ruditapes philippinarum* and differential responses to marine environmental heavy metal contaminants to select one sensitive pedigree of *R. philippinarum* for marine heavy metal pollution biomonitoring and environmental toxicology. In this work, we set out to answer three questions. The answer to the first question is: There were significant biological differences between White and Zebra pedigrees of clams based on the metabolic profiles. The metabolic profiles showed higher levels of alanine, arginine, glutamine, hypotaurine, phosphocholine and homarine in White clam muscles and higher levels of branched chain amino acids (valine, leucine and isoleucine), succinate and 4-aminobutyrate, respectively. The answer to the second question: The metabolic responses were different between White and Zebra clams. The answer to the third question: White clam was more sensitive to Cd exposure based on the sensitive metabolic changes in adductor muscles. Overall, we conclude that White pedigree of clam could be a preferable bioindicator/biomonitor used for marine heavy metal pollution biomonitoring and marine environmental toxicology when adductor muscle is used as the target tissue.

## References

[1] Daviss B (2005). Growing pains for metabolomics. Scientist.

[2] Lindon JC, Nicholson JK, Holmes E, Everett JR (2000). Metabonomics: Metabolic processes studied by NMR spectroscopy of biofluids. Concepts Magn. Reson.

[3] Viant MR, Eric S, Rosenblum ES, Tjeerdema RS (2001). Optimized method for the determination of phosphoarginine in abalone tissue by high-performance liquid chromatography. J. Chromatogr. B.

[4] Brindle JT, Antti H, Holmes E, Tranter G, Nicholson JK, Bethell HWL, Clarke S, Schofield PM, McKilligin E, Mosedale DE (2002). Rapid and noninvasive diagnosis of the presence and severity of coronary heart disease using ^1^H-NMR-based metabonomics. Nat. Med.

[5] Bundy JG, Spurgeon DJ, Svendsen C, Hankard PK, Weeks JM, Osborn D, Lindon JC, Nicholson JK (2004). Environmental metabonomics: Applying combination biomarker analysis in earthworms at a metal contaminated site. Ecotoxicology.

[6] Wu H, Zhang X, Li X, Wu Y, Pei F (2005). Acute biochemical effects of La(NO_3_)_3_ on liver and kidney tissues by magic-angle spinning ^1^H nuclear magnetic resonance spectroscopy and pattern recognition. Anal. Biochem.

[7] Viant MR, Pincetich CA, Hinton DE, Tjeerdema RS (2006). Toxic actions of dinoseb in medaka (*Oryzias latipes*) embryos as determined by *in vivo* ^31^P NMR, HPLC-UV and ^1^H NMR metabolomics. Aquat. Toxicol.

[8] Viant MR, Pincetich CA, Hinton DE, Tjeerdema RS (2006). Metabolic effects of dinoseb, diazinon and esfenvalerate in eyed eggs and alevins of Chinook salmon (*Oncorhynchus tshawytscha*) determined by ^1^H NMR metabolomics. Aquat. Toxicol.

[9] Nicholson JK, Timbrell JA, Sadler PJ (1985). Mercury nephrotoxicity and the detection of abnormal urinary metabolite excretion patterns by high resolution proton nuclear magnetic resonance spectroscopy. Mol. Pharmacol.

[10] Plumb RS, Stumpf CL, Granger JH, Castro-Perez J, Haselden JN, Dear GJ (2003). Use of liquid chromatography/time-of-flight mass spectrometry and multivariate statistical analysis shows promise for the detection of drug metabolites in biological fluids. Rapid Commun. Mass Spectrom.

[11] Wang Y, Bollard ME, Keun H, Antti H, Beckonert O, Ebbels TM, Lindon JC, Holmes E, Tang H, Nicholson JK (2003). Spectral editing and pattern recognition methods applied to high-resolution magic-angle spinning ^1^H nuclear magnetic resonance spectroscopy of liver tissues. Anal. Biochem.

[12] Wu H, Zhang X, Wu Y, Pei F (2005). Studies on the acute biochemical effects of La(NO_3_)_3_ using ^1^H NMR spectroscopy of urine combined with pattern recognition. J. Inorg. Biochem.

[13] Jones OAH, Dondero F, Viarengo A, Griffin JL (2008). Metabolic profiling of *Mytilus galloprovincialis* and its potential applications for pollution assessment. Mar. Ecol. Prog. Ser.

[14] Tuffnail W, Mills GA, Cary P, Greenwood R (2009). An environmental ^1^H NMR metabolomic study of the exposure of the marine mussel *Mytilus edulis* to atrazine, lindane, hypoxia and starvation. Metabolomics.

[15] Wu H, Wang W-X (2010). NMR-based metabolomic studies on the toxicological effects of cadmium and copper on green mussels *Perna viridis*. Aquat. Toxicol.

[16] Tikunov AP, Johnson CB, Lee H, Stoskopf MK, Macdonald JM (2010). Metabolomic investigations of American oysters Using ^1^H-NMR spectroscopy. Mar. Drugs.

[17] Lannig G, Eilers S, Pörtner HO, Sokolova IM, Bock C (2010). Impact of ocean acidification on energy metabolism of oyster, *Crassostrea gigas*—Changes in metabolic pathways and thermal response. Mar. Drugs.

[18] Gordon BR, Leggat W (2010). *Symbiodinium*—Invertebrate symbioses and the role of metabolomics. Mar. Drugs.

[19] Ji J, Choi HJ, Ahn I-Y (2006). Evaluation of Manila clam *Ruditapes philippinarum* as a sentinel species for metal pollution monitoring in estuarine tidal flats of Korea: Effects of size, sex, and spawning on baseline accumulation. Mar. Pollut. Bull.

[20] Laing I, Child AR (1996). Comparative tolerance of small juvenile palourdes (*Tapes decussates* L.) and Manila clams (*Tapes philippinarum* Adams & Reeve) to low temperature. J. Exp. Mar. Biol. Ecol.

[21] Matozzo V, Ballarin L, Marin MG (2004). Exposure of the clam *Tapes philippinarum* to 4-nonylphenol: Changes in anti-oxidant enzyme activities and re-burrowing capability. Mar. Pollut. Bull.

[22] Hegaret H, Da Silva PM, Wikfors GH, Lambert C, de Bettignies T, Shumway SE, Soudant P (2007). Hemocyte responses of Manila clams, *Ruditapes philippinarum*, with varying parasite, *Perkinsus olseni*, severity to toxic-algal exposures. Aquat. Toxicol.

[23] Moraga D, Mdelgi-Lasram E, Romdhane MS, El Abed A, Boutet I, Tanguy A, Auffret M (2002). Genetic responses to metal contamination in two clams: *Ruditapes decussatus* and *Ruditapes philippinarum*. Mar. Environ. Res.

[24] Yan X, Zhang G, Yang F, Liang J (2005). A comparison of growth and development of Manila Clam (*Ruditapes philippinarum*) from two pedigrees (in Chinese). J. Dalian Fish. Univ.

[25] Zhang Y, Yan X, Yao T, Huo Z, Yang F, Zhang G (2008). Study on population hybridization of two shell color strains of Manila clam *Ruditapes philippinarum* (in Chinese). South China Fish. Sci.

[26] Liu X, Zhang L, You L, Yu J, Zhao J, Li L, Wang Q, Li F, Li C, Liu D, Wu H (2011). Differential toxicological effects induced by mercury in gills from three pedigrees of Manila clam *Ruditapes philippinarum* by NMR-based metabolomics. Ecotoxicology.

[27] Mao TY, Dai MX, Peng ST, Li GL (2009). Temporal-spatial variation trend analysis of heavy metals (Cu, Zn, Pb, Cd, Hg) in Bohai Bay in 10 Years (in Chinese). J. Tianjin Univ.

[28] Zhang X (2001). Investigation of pollution of Pb, Cd, Hg, As in sea water and deposit of Bohai Sea area (in Chinese). Heilongjiang Environ. J.

[29] Wu H, Wang W-X (2011). Tissue-specific toxicological effects of cadmium in green mussel (*Perna viridis*): Nuclear magnetic resonance-based metabolomics study. Environ. Toxicol. Chem.

[30] Wu H, Southam AD, Hines A, Viant MR (2008). High throughput tissue extraction protocol for NMR and mass spectrometry based metabolomics. Anal. Biochem.

[31] Lin CY, Wu H, Tjeerdema RS, Viant MR (2007). Evaluation of metabolite extraction strategies from tissue samples using NMR metabolomics. Metabolomics.

[32] Viant MR, Rosenblum ES, Tjeerdema RS (2003). NMR-based metabolomics: A powerful approach for characterizing the effects of environmental stressors on organism health. Environ. Sci. Technol.

[33] Preston RL (2005). Transport of amino acids by marine invertebrates. Comp. Physiol. Biochem.

[34] Liu X, Zhang L, You L, Cong M, Zhao J, Wu H, Li C, Liu D, Yu J (2011). Toxicological responses to acute mercury exposure for three species of Manila clam *Ruditapes philippinarum* by NMR-based metabolomics. Environ. Toxicol. Pharmacol.

[35] Fan WMT (1996). Metabolite profiling by one- and two-dimensional NMR analysis of complex mixtures. Prog. Nucl. Magn. Reson.

[36] Purohit PV, Rocke DM, Viant MR, Woodruff DL (2004). Discrimination models using variance-stabilizing transformation of metabolomic NMR data. OMICS J. Integr. Biol.

[37] Parsons HM, Ludwig C, Gunther UL, Viant MR (2007). Improved classification accuracy in 1- and 2-dimensional NMR metabolomics data using the variance stabilising generalised logarithm transformation. BMC Bioinform.

[38] Xu L (2004). Methods of Chemometrics.

[39] Keun HC, Ebbels TMD, Antti H, Bollard ME, Beckonert O, Holmes E, Lindon JC, Nicholson JK (2003). Improved analysis of multivariate data by variable stability scaling: Application to NMR-based metabolic profiling. Anal. Chim. Acta.

[40] Rubingh CM, Bijlsma S, Derks EPA, Bobeldijk I, Verheij ER, Kochhar S, Smilde AK (2006). Assessing the performance of statistical validation tools for megavariate metabolomics data. Metabolomics.

[41] Lindon JC, Nicholson JK, Everett JR (1999). NMR spectroscopy of biofluid. Ann. Rep. NMR Spectrosc.

[42] Katsiadaki I, Williams TD, Ball JS, Bean TP, Sanders MB, Wu H, Santos EM, Brown MM, Baker P, Ortega F (2009). Hepatic transcriptomic and metabolomic responses in the Stickleback (*Gasterosteus aculeatus*) exposed to ethinyl-estradiol. Aquat. Toxicol.

